# Integrin Signaling in Oligodendrocytes and Its Importance in CNS Myelination

**DOI:** 10.1155/2011/354091

**Published:** 2010-12-20

**Authors:** Ryan W. O'Meara, John-Paul Michalski, Rashmi Kothary

**Affiliations:** ^1^Regenerative Medicine Program, Ottawa Hospital Research Institute, 501 Smyth Road, Ottawa, ON, Canada K1H 8L6; ^2^Department of Cellular and Molecular Medicine, University of Ottawa, Ottawa, ON, Canada K1H 8M5; ^3^Department of Medicine, University of Ottawa, Ottawa, ON, Canada K1H 8M5

## Abstract

Multiple sclerosis is characterized by repeated demyelinating attacks of the central nervous system (CNS) white matter tracts. To tailor novel therapeutics to halt or reverse disease process, we require a better understanding of oligodendrocyte biology and of the molecular mechanisms that initiate myelination. Cell extrinsic mechanisms regulate CNS myelination through the interaction of extracellular matrix proteins and their transmembrane receptors. The engagement of one such receptor family, the integrins, initiates intracellular signaling cascades that lead to changes in cell phenotype. Oligodendrocytes express a diverse array of integrins, and the expression of these receptors is developmentally regulated. Integrin-mediated signaling is crucial to the proliferation, survival, and maturation of oligodendrocytes through the activation of downstream signaling pathways involved in cytoskeletal remodeling. Here, we review the current understanding of this important signaling axis and its role in oligodendrocyte biology and ultimately in the myelination of axons within the CNS.

## 1. Introduction

Multiple sclerosis (MS) is an inflammatory disease of the CNS resulting in physical and cognitive disabilities. Repeated attacks to the myelin sheath result in its degeneration [1], and over time, these demyelinating events leave sclerotic plaques that are resistant to remyelination. Oligodendrocytes are the myelin-producing cells of the CNS, providing the insulation of axons that facilitates efficient saltatory conduction of action potentials down the neuron.

Oligodendrocytes originate from neuroepithelial cells as oligodendrocyte precursor cells (OPCs) from the subventricular region of the embryonic brain and ventral spinal cord [2]. OPCs have simple morphology relative to their differentiated state, bearing only a few processes. These cells are highly responsive to soluble growth factors (GFs), which promotes their proliferation/survival [3]. As OPCs mature, they begin to extend a complex meshwork of processes with the goal of contacting multiple axons. Once axo-glial contact has been established, oligodendrocytes produce large amounts of specialized membrane (myelin) that form multiple wraps around the contacted axons. The final stages of myelination involve the expulsion of cytoplasm from the membrane wraps to form compact myelin. The molecular events underlying oligodendrocyte proliferation, survival, and maturation are poorly understood, yet knowledge of these processes is of great value for the development of therapeutics for demyelinating diseases like MS.

Several signaling pathways have been implicated in the sequence of molecular events leading to oligodendrocyte-mediated myelination. It is clear that functional interaction between integrins expressed by oligodendrocytes and laminin expressed on the cell surface of the neurons they support is essential for proper myelination to occur. Much research has been directed towards the integrin family of receptors and their impact on various aspects of oligodendrocyte biology. Oligodendrocytes express a specific set of developmentally regulated integrin receptors. Integrins are responsible for mediating a connection between the extracellular matrix (ECM) and actin cytoskeleton, thus relaying signals between both compartments to govern various cellular processes. Specifically, integrin signaling has been linked to GF-mediated survival and proliferation of oligodendrocytes. Integrin-based amplification of GF-signaling depends on common proliferation/survival pathways involving phosphoinositide 3-kinase (PI3K) and mitogen-activated protein kinase (MAPK) cascades. PI3Ks are a family of ubiquitously expressed proteins responsible for a broad variety of cellular functions including signal transduction, migration, proliferation, survival, and cytoskeletal reorganization [4]. Likewise, MAPKs are a group of signaling proteins which form part of a signaling cascade regulating diverse functions such as differentiation, proliferation, and apoptosis [5].

 Oligodendrocyte differentiation and maturation are also impacted by integrin signaling, involving proteins such as Fyn kinase. In response to integrin engagement, Fyn kinase activates various downstream signaling pathways, including small GTPases such as Cdc42, Rac1, and Rho. Known for their roles in cytoskeletal remodeling, these small GTPases respond to integrin signaling to govern the morphological changes of oligodendrocytes. The goal of the present paper is to outline the importance of integrin signaling in various aspects of oligodendrocyte biology.

## 2. Oligodendrocyte Expression of Integrins

Accumulating evidence suggests that ECM proteins regulate various aspects of oligodendrocyte biology. Specifically, ECM constituents such as fibronectin (FN), vitronectin (VN), and laminin-2 (LN2) have been associated with oligodendrocyte proliferation, survival, and development [6–18]. These ECM proteins bind to integrin family members, and mediate cellular adhesion and bidirectional signaling between the cellular interior and the external environment. Integrin receptors are heterodimeric structures composed of *α* and *β* subunits. Eight *β* subunits along with 18 *α* subunits have been discovered, with one *α* subunit partnering with one *β* subunit to form the 24 known integrin heterodimer receptors [19]. The *α* subunits are believed to specify integrin complexes to specific ECM components. For example, *α*6-integrin tends to specify the *β*1 subunit to laminins, whereas *α*v subunits specify integrins to RGD-containing ligands such as FN and VN [19]. Oligodendrocytes express *α*v subunit complexed with *β* subunits 1, 3, 5, and 8 [20, 21]. These cells also express the *α*6 subunit, however they do not express *α* subunits 1–5 [20]. 

**Figure 1:**
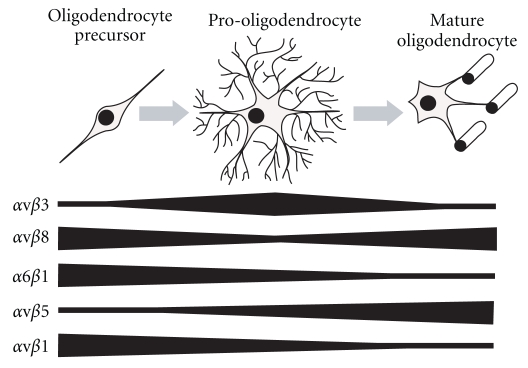
Representation of integrin receptor expression over the course of oligodendrocyte development. Oligodendrocytes originate as precursor cells with simple morphology and subsequently differentiate into pro-oligodendrocytes, characterized by extension of intricate process meshworks. The final stages of maturation involve wrapping of axons in multiple layers of myelin membrane. Integrin receptors are differentially expressed during oligodendrocyte maturation. *α*v*β*1-integrin and *α*6*β*1-integrin are strongly expressed in the oligodendrocyte precursor phase, whereas *α*v*β*5-integrin is strongly expressed in late stages of development. *α*v*β*8 is principally expressed in early and late stages of oligodendrocyte maturation, while *α*v*β*3-integrin is expressed strongly in intermediate stages.

Expression of integrins is developmentally regulated, and different cell types express diverse arrays of integrins depending on their stage of differentiation. Oligodendrocytes are no exception, variably expressing *α* and *β* subunits based on developmental stage (Figure 1). For example, *α*v*β*8-integrin is expressed strongly by precursor and mature oligodendrocytes, whereas *α*v*β*3-integrin is expressed predominantly during intermediate maturity stages [21]. Newly formed oligodendrocytes strongly express *α*6*β*1-integrin, whereas *α*v*β*5 expression increases as oligodendrocytes mature [20, 22, 23]. 

The expression pattern of integrins by oligodendrocytes is not only developmentally regulated, but is affected by the nature of the surrounding environment. Changes in the ECM constituents are reflected by alterations in oligodendrocyte integrin expression. For example, when oligodendrocyte-like cells derived from hippocampal neural progenitors are seeded on LN2, *β*1-integrin expression is increased with respect to cells seeded on non-LN2 substrates [24]. The presence of cultured neurons also appears to influence the expression profile of OL integrin receptors. Milner et al. [21] observed that the decrease in expression of *α*v*β*8-integrin in isolated oligodendrocytes during intermediate stages of development is less pronounced when cocultured with neurons.

The above studies highlight the dynamic nature of integrin expression by oligodendrocytes and allow for further questioning the roles played by integrin receptors in oligodendrocyte biology. Generally, integrins mediate diverse cellular functions of oligodendrocytes, especially proliferation, survival, differentiation, and maturation.

## 3. Proliferation and Survival of Oligodendrocytes

During maturation, oligodendrocytes experience increased expression of proapopotic molecules, whereas antiapoptotic protein activity remains unchanged [25]. Oligodendrocytes are thus especially susceptible to programmed cell death during development, highlighting the importance of extracellular cues for promoting their proliferation/survival. Classical studies on oligodendrocyte growth and survival have demonstrated roles for both PI3K and MAPK pathways in various aspects of oligodendrocyte biology [26, 27]. More recently, these pathways appear to be impacted by integrins, mediated through their interaction with ECM molecules.

Integrin ligation by ECM proteins appears to be a key factor in the proliferation and survival of oligodendrocytes. In parallel, trophic factors such as platelet-derived growth factor (PDGF) and neuregulin-1 (NRG) also support oligodendrocyte proliferation and survival [28]. Recently, integrins and GF receptors (GFRs) have been shown to cooperate in a fashion to promote proliferation and survival in response to various ECM and trophic signals. These signals are subsequently relayed to downstream signaling molecules such as PI3K and MAPK, principally via Src family kinases (SFKs). To fully understand the regulation of oligodendrocyte proliferation and survival, the relationship between integrin ligation and GF reception must be explored. The following sections will cover some key studies highlighting advances in the knowledge of integrin signaling in oligodendrocyte proliferation and survival.

### 3.1. Integrin Activation Sensitizes GF Signaling

The importance of integrin receptors in oligodendrocyte proliferation and survival is evidenced by a number of studies involving genetic manipulation of integrin receptor subunits. For example, the expression of a dominant-negative *β*1-integrin in oligodendrocytes causes a region-specific depletion of mature (CC1+) oligodendrocytes in certain CNS regions, followed by alterations in MAPK activity [29]. *β*1-integrin subunit blocking also dramatically decreases oligodendrocyte survival [7], and deletion of the *β*1-integrin gene in murine oligodendrocytes leads to oligodendroglial apoptosis within the embryonic brainstem [30]. *α*6-integrins also play a role in proliferation/survival, since *α*6-integrin null mice have increased apoptosis of newly formed oligodendrocytes [10]. This work indicates a clear role for integrin receptors in oligodendrocyte survival, and the mechanism governing integrin-enhanced survival is thought to involve facilitation of GF signaling. 

While integrin-based adhesion in itself does not promote oligodendrocyte survival, integrin ligation with ECM substrates influences GFR-based survival signaling. This phenomenon has been observed in a number of studies, where the *α*6*β*1-integrin substrate, LN2, increases the sensitivity of oligodendrocytes to soluble PDGF thereby facilitating survival [7, 10, 17, 31, 32]. The *α*6*β*1-integrin is the only laminin receptor of the integrin family expressed by oligodendrocytes [20] suggesting that *α*6*β*1-integrin is responsible for LN2-mediated enhancement GF survival signaling. Expression of chimeric *α*-integrin subunits in oligodendrocytes further supports this hypothesis [10]. Under normal conditions, the *α*6*β*1-integrin receptor binds LN2, while the integrin *α*5-containing receptors preferentially bind FN. In the latter study, the extracellular and transmembrane domains of *α*5-integrin were fused with the cytoplasmic tail of the *α*6-integrin subunit. Expression of this hybrid *α*5/*α*6-integrin subunit in oligodendrocytes increased survival during concurrent FN adhesion and PDGF administration, a response normally observed on LN2 substrate via *α*6*β*1-integrin receptors. These results indicate that the survival-promoting events occurring via concurrent LN2 ligation and PDGF administration are mediated via *α*6*β*1-integrin receptors. While this study demonstrates a relationship between integrin and PDGF signaling, it does not explain how integrins and GFRs (such as PDGFR-*α*) interact. More recent research has aimed to determine the mechanism by which integrins interact with other receptor types to promote oligodendrocyte proliferation and survival [9, 10, 13, 17, 33].

### 3.2. Integrins and GFR Interactions

How does the cooperation between integrins and GFRs promote survival in oligodendrocytes? A leading hypothesis suggests that GFRs and integrin receptors are recruited to one another during concurrent integrin and GFR ligation. In light of this hypothesis, *α*v*β*3-integrin has been described to physically interact with PDGFR-*α* in oligodendrocytes grown on VN (*α*v*β*3-integrin ligand), and in the presence of PDGF [9]. Oligodendrocyte proliferation under these conditions is perturbed by blocking the PI3K and PKC signaling pathways, suggesting *α*v*β*3/PDGFR-*α* signaling is dependant on this pathway. A similar result was obtained by Colognato et al. [12], where *α*v*β*3-integrin-enhanced PDGF oligodendrocyte signaling was impaired with knockdown of the SFK family member Fyn. 

**Figure 2:**
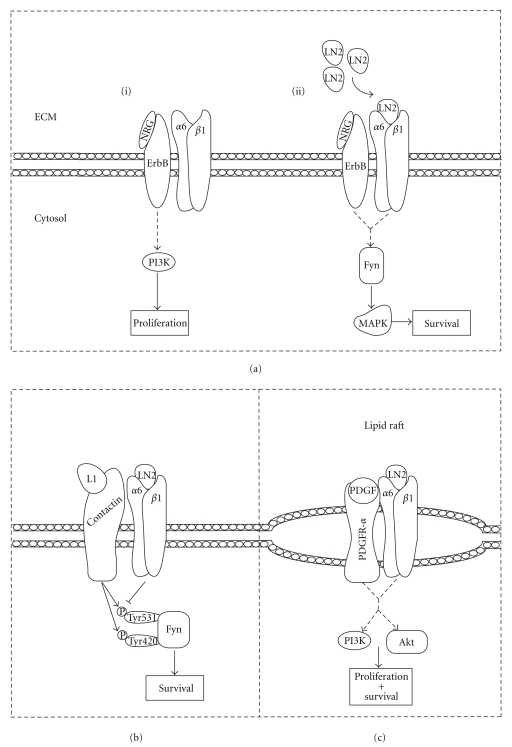
Integrins interact with other receptor types to mediate oligodendrocyte survival and proliferation. (a) Cooperative signaling between ErbB and integrin receptors. Binding of NRG to oligodendrocyte ErbB receptors normally evokes a proliferative response dependant on PI3K signaling. Upon *α*6*β*1-integrin ligation with LN2, NRG binding no longer induces PI3K activation, but rather initiates the MAPK survival response via Fyn signaling. (b) Joint regulatory control of survival in response to cooperative contactin and integrin receptor signaling. LN2 ligation of *α*6*β*1-integrin causes the dephosphorylation of Fyn's negative regulatory site Tyr531. L1 binding to the contactin receptor induces the phosphorylation of Tyr420 (activation site) and Tyr531. Integration of extracellular cues via contactin/integrin crosstalk facilitates the dynamic control of Fyn to execute cellular functions affecting survival. (c) PDGFR-a and *α*6*β*1-integrin interact in oligodendrocyte lipid rafts. PI3K and Akt are activated upon concurrent ligation of PDGFR-*α* and *α*6*β*1-integrin with PDGF and LN2, respectively, to promote proliferation/survival.

Oligodendrocyte integrin receptors also modulate signaling by GFs other than PDGF, such as neuregulin-1 (NRG) [10, 12, 28, 34]. NRG is a soluble GF with a role in oligodendrocyte proliferation and survival [28]. NRG is predominantly expressed on the neuronal surface and acts as a survival and proliferative cue for oligodendrocytes through ErbB receptors [34]. On non-laminin substrates, NRG application to oligodendrocytes induces their proliferation via the PI3K pathway (Figure 2(a)[i]) [10]. Under these conditions, oligodendrocytes are sensitive to wortmannin treatment and thus depend on the PI3K pathway for survival. Once oligodendrocytes are seeded on LN2 (*α*6*β*1-integrin ligand), they lose their sensitivity to wortmannin, indicating an altered function of NRG. On LN2 rather, NRG activates the MAPK survival pathway (Figure 2(a)[ii]), as evidenced by increased phosphorylation (and thus inactivation) of the apoptotic protein BAD. Also, knockdown of SFK member Fyn prevents LN2-induced switching of the PI3K/MAPK pathways in response to soluble NRG [12]. These studies suggest that *α*6*β*1-integrin ligation mediates a switch in the function of NRG; without LN2 binding, NRG promotes a proliferative signal via PI3K. Conversely, upon LN2 adhesion, NRG no longer promotes proliferation but rather promotes differentiation/survival via MAPK, and Fyn is required for this signaling transition. Since certain laminin subunits are expressed on axons of the CNS [35–37], integrin-mediated adhesion of oligodendrocytes to axonal laminins may amplify NRG signaling via Fyn to promote differentiation/survival over proliferation. This enhanced survival is specifically mediated by *α*6*β*1-integrin and is also observed during oligodendrocyte contact with astrocytic laminins [38].

Integrin receptors may also interact with non-GFRs to mediate adhesion-based survival enhancement. In a recent report from Laursen et al. [17], oligodendrocyte *α*6*β*1-integrins were shown to physically interact with the non-GF receptor contactin. Contactin is the receptor for the axonally expressed L1 ligand [39, 40] and may play a role in myelination. As previously observed, oligodendrocytes grown on LN2 showed a dose-dependant increase in survival in response to physiological doses of PDGF. This effect was also observed when cells were grown on non-LN2 substrates in the presence of L1-Fc and PDGF. Culturing of cells on LN2 with L1-Fc and PDGF proved to have an additive effect to promote survival, suggesting a synergistic relationship between integrin, contactin and PDGFR-*α* signaling. This cooperative signaling in response to ligation influences the phosphorylation state of Fyn kinase. Fyn may be phosphorylated at Tyr420 (activation site) or at Tyr531 (inhibitory site), which allows for dynamic dual regulatory control of Fyn activity [41]. Upon binding LN2, *α*6*β*1-integrin ligation causes the dephosphorylation of Tyr531, while contactin/L1 binding increased phosphorylation of both Tyr531 and Tyr420 sites [17]. Ligation with either LN2 or L1 partially enhanced survival, whereas copresentation of both ligands further intensified the potential for survival. This provides joint regulatory control of oligodendrocyte survival signaling via Fyn in response to the axonal surface and surrounding ECM (Figure 2(b)). In order for these signaling events to occur efficiently, there must be coordination between individual proteins within the integrin signaling pathway. In oligodendrocytes, this compartmentalization of signaling cascades likely involves lipid rafts.

### 3.3. Lipid Rafts Stabilize GFR and Integrins

Lipid rafts are membrane structures composed of embedded lipoproteins and signaling molecules such as Fyn [42]. These structures are more organized than the typical bilayer, but are free floating within it. They tend to contain more cholesterol and have increased levels of glycosphingolipids such as sphingomyelin [43, 44]. Lipid rafts ensure that signaling cascade constituents are within appropriate molecular distance to facilitate signal transduction by raft-bound receptors [45]. 

During development, oligodendrocytes extend membrane sheets eventually forming myelin, facilitating neural transmission. The biochemistry of these specialized membrane sheets must be critical for their function, likely requiring lipid raft-mediated signaling for myelin biogenesis. In oligodendrocytes, integrins rely on PI3K and Akt for the transduction of ECM-derived signals. PI3K recruitment to lipid rafts occurs during growth of oligodendrocytes on LN2, a known *α*6*β*1-integrin ligand [13]. *α*6*β*1-integrin also localizes to oligodendrocyte lipid rafts, and raft disruption via chemical means perturbs PI3K-dependant *α*6*β*1-integrin mediated survival. Blocking of the *β*1-integrin subunit also depletes phosphorylated Akt within rafts. Therefore, *α*6*β*1-integrin receptors are localized within oligodendrocyte lipid rafts, and activate downstream signaling proteins to enhance survival in response to LN2 ligation. Integrin function is important in oligodendrocyte lipid rafts, but how do integrins interact with GFRs within these structures to amplify survival signaling?

For efficient interaction between integrins and GFRs to occur, it is fair to assume that they must be located/recruited within close proximity to each other. Research has suggested that the mechanism underlying GFR/integrin cooperative signaling is facilitated by colocalization of these receptors within lipid rafts as shown by Baron et al. [33]. In this study, PDGFR-*α* localized to oligodendrocyte lipid rafts. Baron [33] also demonstrated oligodendrocyte growth on LN2 substrate with soluble PDGF recruits PDGFR-*α* to *α*6*β*1-integrin rich rafts, and *α*6*β*1-integrin/PDGFR-*α* clustering accelerated the phosphorylation of Akt and PI3K within lipid rafts (Figure 2(c)).

Similar phenomena occur in other cell systems such as keratinocytes, where clustering of *α*6*β*4-integrin with epidermal growth factor receptor is required for GF-dependant proliferation via SFK signaling [46]. In terms of oligodendrocytes, cooperative signaling between lipid raft-localized integrins and PDGFR-*α* influence PI3K and Akt signaling which are important for proliferation and survival. Of equal importance are the signaling cascades underlying oligodendrocyte differentiation and myelination, processes also governed by the integrins.

## 4. Differentiation and Myelination

Integrin receptors are important for the normal morphological development of oligodendrocytes *in vitro*, and for their myelinating function *in vivo*. Of the various integrin receptors expressed by oligodendrocytes, the *α*6*β*1-integrin is the only known integrin complex that binds LN2 [19, 20]. Since many axonal tracts of the CNS express laminins, it is reasonable to hypothesize that *α*6*β*1-integrin plays an important role in myelination [35–37]. Specifically, it appears the *β*1 subunit of the *α*6*β*1 receptor is especially crucial for oligodendrocyte maturation; *β*1-integrin relays signals from the ECM to downstream signaling proteins ultimately impacting on development. Intracellular proteins that have been recently associated with *β*1-integrin signaling include integrin linked kinase (ILK), focal adhesion kinase (FAK), Akt, Fyn, Rac1, Cdc42, and Rho. These proteins are largely involved in cytoskeletal remodeling and therefore may play an important role in the morphological development of oligodendrocytes in response to *β*1-integrin activation.

### 4.1. *β*1-Integrin in Oligodendrocyte Development

The influence of integrins on oligodendrocyte development is thought to be governed by ECM components in axonal tracts [10]. Disruption of ECM-integrin connection results in a variety of developmental defects, including aberrant process and myelin sheet formation, along with delayed expression of myelin proteins. In contrast, integrin ligation facilitates the normal development of oligodendrocytes, as illustrated by work showing that oligodendroglial membranes are enhanced upon constitutive integrin activation [47]. Membrane extension *in vitro* appears to be largely impacted by *β*1-integrin ligation. Antibody blocking of *β*1-integrin reduces the ability of oligodendrocytes to extend processes *in vitro*, leading to a less arborous morphology as compared to control-treated cells [6]. Parallel results replicated by several other groups highlight the importance of *β*1-integrin signaling in normal oligodendrocyte development [14, 32, 48]. Even perturbation of the *β*1-integrin binding partner ILK is sufficient to cause oligodendrocyte morphological defects [11]. 

The question of how *β*1-integrin impacts oligodendrocyte function *in vivo* is a topic of recent debate [29–32, 48, 49]. The controversy may be caused by a combination of three main factors; the alternate methods used to disrupt *β*1-integrin, the varying promoters used to elicit *β*1-integrin perturbation and/or the existence of non-integrin type receptors with a role in myelination. Firstly, studies use different promoters to drive the ablation/mutant overexpression of *β*1-integrin. The use of alternative promoters may affect the specificity of expression to oligodendrocytes, as well may vary the developmental time during which this expression is incurred. Benninger et al. [30] observed no obvious defects in CNS myelination when *β*1-integrin was ablated in oligodendrocytes under the 2′,3′-cyclic nucleotide 3′-phosphodiesterase (CNP) promoter. CNP is expressed during later stages of oligodendrocyte development [50], therefore use of this promoter may preclude expression during early oligodendrocyte differentiation. Perhaps *β*1-integrin expression during these early stages is crucial for the initiation of myelination, and ablation ensuing this critical window may produce no overt myelin defect. To address this possibility, Barros et al. [32] used a nestin promoter-driven Cre recombinase to ablate *β*1-integrin. Nestin is expressed in neurons and glia of the CNS, thus, use of this promoter would ablate *β*1-integrin early in oligodendrocyte development [51]. In this study, abnormal myelination was observed in the optic nerve, cerebellum, and spinal cord [32], emphasizing the importance of promoter choice when perturbing *β*1-integrin.

The second possible explanation for the controversy surrounding the importance of *β*1-integrin in CNS myelination involves the contrasting means of disrupting *β*1-integrin among studies (i.e., various dominant-negative constructs versus complete ablation). However, it is important to remember that this second possibility remains a function of the promoter used to drive mutant expression. Early work demonstrated a reduced ability of dominant-negative *β*1-integrin expressing oligodendrocytes to remyelinate axons in acutely demyelinated rodent nerve tracts [48]. Myelination defects were also seen when Lee et al. [29] overexpressed a dominant negative version of *β*1-integrin lacking the C-terminal cytoplasmic tail under the proteolipid protein (PLP) promoter. The latter group observed hypomyelination in the spinal cord and optic nerves, which is in contrast to the study by Benninger et al. [30], who used complete ablation over the dominant-negative approach. The differing results may be explained by the alternative means used to disrupt *β*1-integrin (i.e., dominant-negative versus knockout), or, as mentioned above, the different promoters used to drive the mutation (PLP versus CNP). This highlights the importance for a consensus on which promoter to use when driving mutation expression in oligodendrocytes, and which dominant-negative/knockout model to take advantage of when disrupting *β*1-integrin.

The third possible source of discrepancy may be explained by the existence of another type of oligodendrocyte laminin receptor with a role in myelination. *α*6*β*1-integrin is the only known laminin receptor of the integrin family expressed by oligodendrocytes [20]. Of interest, the dystroglycan receptor has been recently identified as a second laminin receptor with a role in CNS myelination. Depletion of dystroglycan causes a reduced ability of oligodendrocytes to form myelinated segments on neurons in an oligodendrocyte/neuron coculture [31]. In fact, *β*1-integrin and dystroglycan have overlapping laminin binding regions and thus may compete for this ligand *in vivo* [52, 53]. Since the dominant-negative *β*1-integrin used by Lee et al. [29] is hypothesized to retain laminin binding, Câmara et al. [49] argues these mutant integrin receptors may compete with dystroglycan for the laminin ligand. This would result in an apparent mutant *β*1-integrin-mediated reduction of myelin thickness. However, the underlying cause of the hypomyelination may be due to fewer occupied dystroglycan receptors, which are required for myelin maintenance [31]. To resolve the possible competitive effect, Câmara et al. [49] expressed a chimeric *β*1-integrin subunit consisting only of its cytoplasmic tail specifically in oligodendrocytes. This dominant-negative *β*1-integrin should be unable to form laminin receptors when bound to *α* subunits, thus eliminating possible competition with dystroglycan. While they observed no overt myelination defects upon expression of this mutant *β*1-integrin receptor, closer examination revealed that larger-diameter axons were preferentially myelinated over smaller-diameter axons. In this report, Câmara et al. [49] discusses possible mechanisms on how this may occur. First, oligodendrocytes in small-caliber axon tracts are required to extend more processes than oligodendrocytes in large axon fiber tracts, since more axons exist per cross-sectional area in small-caliber axon tracts. Since *β*1-integrin perturbation hinders process extension, this debility would prevent oligodendrocytes from extending membrane to contact neighboring axons. This defect in axo-glial contact would be exacerbated in regions of small-caliber axons due to the larger number of axons with respect to large-caliber axon tracts [49]. 

Another possible reason for the reduced myelination of small axons upon *β*1-integrin perturbation involves trophic signaling [49]. LN2 has a promyelinating influence on oligodendrocytes, which is mediated through *β*1-integrin. This promyelinating effect is proportional to axonal caliber. Therefore, the increased amount of promyelinating LN2 ligand on large-caliber axons may allow for *β*1-integrin deficient oligodendrocytes to overcome the defects seen in regions of smaller-caliber axons [49]. 

Based on the evidence presented in the above-mentioned studies, perturbation of *β*1-integrin results in abnormal CNS myelination under certain conditions. More recent work aims to determine which downstream signaling proteins may underlie the defects observed in *β*1-integrin deficient oligodendrocytes. Culminating evidence implicates signaling proteins such as Fyn, Akt, FAK, and small GTPases.

### 4.2. Integrin Influence on Cytoskeletal Remodeling Proteins

**Figure 3:**
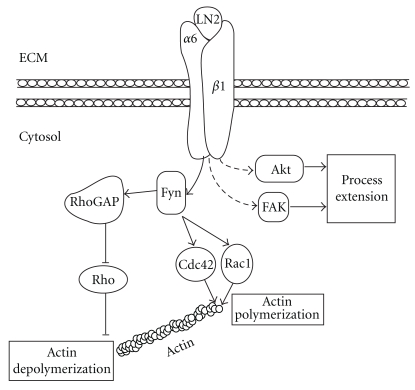
The influence of *α*6*β*1-integrin signaling on oligodendrocyte morphology. Signaling proteins FAK and Akt are phosphorylated upon LN2-mediated activation of *α*6*β*1-integrin. Similarly, Fyn is activated via *α*6*β*1-integrin binding to LN2, thereby activating the cytoskeletal remodeling proteins Rho, Cdc42, and Rac1. The activation of FAK, Akt, Rho, Cdc42, and Rac1 by *α*6*β*1-integrin signaling impacts on oligodendrocyte process extension and is likely implicated in oligodendrocyte development. Solid and dashed lines, respectively, represent direct and indirect interactions.

Integrin activation via extracellular ligands has been investigated in a wide variety of cell types, and is known to result in recruitment of signaling proteins to focal adhesion sites. FAK and Akt are two such proteins and have been extensively studied with regards to their roles in oligodendrocyte biology and myelination [15, 18, 54–61]. These integrin-associated proteins are believed to transduce signals from the cytoplasmic domain of *β*1-integrin. FAK is a protein tyrosine kinase involved in cellular adherence and spreading, whereas Akt is a serine/threonine protein kinase, and its activity promotes cellular survival (Figure 3). Although it is not clear whether FAK or Akt directly interacts with *β*1-integrin in oligodendrocytes, evidence suggests they are activated by integrins within focal adhesions [33, 62–64]. Specifically, the Tyr397 residue of FAK undergoes autophosphorylation during integrin ligation by ECM components [65]. This occurs in oligodendrocytes, where phosphorylation of FAK at various tyrosine residues (including Tyr397) is enhanced when cells are seeded on LN2 substrate [15]. Similarly, Akt phosphorylation within *α*6*β*1-integrin rich lipid rafts is increased in response to concurrent LN2 ligation and PDGF administration [33]. Increased FAK and Akt activation in response to LN2 binding suggests *β*1-integrin mediates this signaling process. Since early *in vitro* work demonstrates oligodendrocyte morphological defects upon depletion of *β*1-integrin [6, 14, 32, 48], does altered expression of FAK and Akt impact on oligodendrocyte development? 

In fact, knockdown of FAK prevents oligodendrocyte process extension in response to LN2 adhesion [15, 18]. This LN2-specific developmental defect upon knockdown of FAK reinforces the notion that *β*1-integrin signaling in maturing oligodendrocytes is required for normal morphological development. Process extension defects are also seen in oligodendrocytes where Akt signaling is perturbed. Upon depletion of *β*1-integrin in oligodendrocytes, Akt activity is consequently reduced. The resultant reduction in Akt activity likely underlies the developmental defects observed in these *β*1-integrin deficient oligodendrocytes [32]. This hypothesis is further supported by Flores et al. [57] and Narayanan et al. [60]. Upon expression of constitutively active Akt, the CNS is hypermyelinated [57, 60] suggesting that overstimulation of the Akt signaling pathway produces overdevelopment of oligodendrocytes, the opposite of what is seen upon reduction of Akt activity.

 Interestingly, conditional knockout of FAK during the onset of myelination results in a reduction in the number of myelinated axons [59]. This is compensated for at later time points, but regardless, both FAK and Akt appear to be important players in transducing ECM signals via *β*1-integrin to contribute to oligodendrocyte development.

Other studies have implicated Fyn in integrin signaling, ultimately affecting oligodendrocyte development. In contrast to FAK and Akt, Fyn is known to directly associate with *α*6*β*1-integrin in oligodendrocytes under various GF and substrate conditions [12, 42]. Early work showed oligodendrocyte precursors express Fyn, and Fyn's expression increases as oligodendrocytes mature [42, 66]. Both pharmacological inhibition and mutagenesis studies demonstrate a requirement for Fyn in membrane sheet formation in primary oligodendrocytes [66]. In addition to morphological defects upon Fyn knockdown, Colognato et al. [12] reported a reduction in MBP-expressing cells, suggesting the expression of crucial myelin proteins is also dependant on Fyn activity. The presence of LN2 also highly affects Fyn's activity, and in laminin-deficient mice (*dy/dy*) there is an increase in the phosphorylation of Fyn's negative regulatory site (Tyr531). The above studies lend evidence to a linear signal cascade initiated by *α*6*β*1-integrin ligation by LN2, and subsequent signal transduction via Fyn to activate downstream signaling proteins. Thus, it is of interest to determine which proteins Fyn is effectuating to ensure normal oligodendrocyte development. Recently, small GTPases such as Cdc42, Rac1, and Rho appear to provide this role.

Cdc42, Rac1, and Rho are GTPases expressed by oligodendrocytes [14, 67], among other cell types. GTPases are considered active when bound to GTP, and inactive when GDP-bound. GTP-bound Cdc42 and Rac1 build filamentous actin while GTP-bound Rho depolymerizes actin filaments, thus, these proteins impact on cell morphology. Integrin signaling has proven to play a role in GTPase-mediated oligodendrocyte morphological differentiation. For example, Fyn activation via *α*6*β*1-integrin ligation influences the activity of Rac1, Cdc42, and Rho (Figure 3). Wang et al. [68] showed that depletion of activated Fyn in oligodendrocytes depletes GTP-bound Rac1, GTP-bound Cdc42, and GDP-bound Rho. Consequently, this altered GTPase activity accompanied morphological defects, offering an importance for these proteins in oligodendroglial process extension [68]. In addition, Fyn phosphorylates RhoGAPs, which are responsible for modulating the GDP-bound state of Rho. Fyn phosphorylates p190RhoGAP, and this phosphorylation event increases with oligodendrocyte differentiation [69]. The activity of Rho heavily influences oligodendrocyte development, since studies using dominant-negative Rho (mimics GDP-bound state) reported hyperextension of oligodendrocyte processes [69]. 

Liang et al. [14] provided more evidence showing that Cdc42 and Rac1 are activated by Fyn in a *β*1-integrin-dependant manner. This group made use of various experimental paradigms to show that *β*1-integrin influences the phosphorylation state of Fyn, ultimately impacting oligodendrocyte morphology via Cdc42 and Rac1 activity. Oligodendrocyte process extension was reduced when cells were treated with *β*1-integrin blocking antibodies, as well as kinase dead or non-palmitoylable/myristoylable Fyn. These Fyn constructs either possessed no kinase activity (kinase dead) or were unable to localize to the plasma membrane (non-palmitoylable/myristoylable), where Fyn is theorized to be activated. The application of *β*1-integrin blocking antibodies also directly reduced Fyn's activity as measured by autophosphorylation assays, and prevented the activation of Cdc42 and Rac1 [14]. The importance of these GTPases in myelination was further evidenced by Thurnherr et al. [70] who specifically ablated Cdc42 or Rac1 in oligodendrocytes *in vivo*. Ablation of either Cdc42 or Rac1 resulted in aberrant myelin outfoldings, whereas the double Cdc42/Rac1 knockout exacerbated this phenotype. Therefore, Cdc42 and Rac1 likely work synergistically to modulate similar effectors of a common pathway to mediate myelin sheath formation. 

From the above studies, a number of conclusions can be drawn. First, it is clear that *β*1-integrin ligation by LN2 influences the activity states of FAK and Akt. This change in activity of FAK and Akt impacts on oligodendroglial differentiation as evidenced by altered capacity to extend processes. Second, Fyn is directly activated by ligated *β*1-integrins, and Fyn influences the activity state of Cdc42 and Rac1 (Figure 3). Once activated by Fyn, these proteins modulate actin dynamics, ultimately governing oligodendrocyte morphology. Misregulation of the aforementioned signaling proteins results in aberrant oligodendrocyte development both *in vitro* and *in vivo*. Therefore, *β*1-integrin signaling to downstream effectors is required for normal oligodendrocyte development.

### 4.3. Integrin Influence on CNS Remyelination

The involvement of the ECM and integrin signaling cascades in oligodendrocyte development and myelination raises the question of whether these systems play a role in remyelination of the CNS after demyelination is induced in diseases such as MS. Demyelination is characterized by the progressive loss of myelin surrounding axons, negatively impacting on propagation of neuronal impulses. Demyelinated lesions can be remyelinated by adult OPCs; however, over time sclerotic plaques become resistant to this process. While the cause of remyelination resistance is unknown, it is likely not due to a lack of ECM molecules required for oligodendrocyte-mediated remyelination. Evidence supporting this possibility is provided by Satoh et al. [71], who conducted proteome profiling on postmortem sclerotic lesions obtained from MS patients. This analysis indicated an enrichment of integrin, focal adhesion, and ECM-related proteins in various types of MS plaques. In parallel, using animal demyelination/remyelination models, some studies demonstrate an increased deposition of ECM constituents following demyelination of white matter tracts. Of interest, Zhao et al. [72] observed increased LN2 expression in axonal tracts during formation of new myelin sheaths following ethidium bromide-induced demyelination. This is similar to the observation of ECM molecules on demyelinated axon processes within MS-patient derived plaques [73]. These studies suggest that ECM ligands necessary for integrin-mediated remyelination are present in demyelinated lesions, but they do not address whether the interaction of oligodendrocyte integrins with these ligands is important for remyelination.

Relvas et al. [48] demonstrated an importance for oligodendrocyte *β*1-integrin in the remyelination of rodent spinal cords. This group transplanted OPCs expressing a dominant-negative *β*1-integrin construct into demyelinated spinal cord lesions. Relative to OPCs expressing a vector control, the OPCs expressing the dominant-negative *β*1-integrin were unable to remyelinate an equivalent number of axons over a three week period [48]. In a cuprizone demyelination paradigm, Lee et al. [29] demonstrated a decrease in total number of remyelinated axons in the corpus callosum of mice expressing an oligodendrocyte-specific dominant-negative *β*1-integrin. Additionally, the *α*-integrin subunit has also been shown to play a role in remyelination. Subsequent to toxin-induced focal demyelination of the rat caudal cerebellar peduncle, there is an upregulation of *α*v-integrin in OPCs within the lesion site [72].

It is apparent that ECM molecules are expressed in lesions following demyelination, and integrin function plays a role in the remyelination process. The obvious question is, why do plaques become resistant to remyelination following repeated demyelination? The failure of sclerotic plaques to remyelinate over time may be due to the accumulation of myelin debris within the plaques themselves. Myelin debris inhibits the differentiation of OPCs, thereby rendering the plaques resistant to remyelination. Research has shown that modulation of downstream proteins involved in integrin signaling potentiates the differentiation of OPCs, even in these remyelination-resistant conditions. Specifically, manipulation of the Fyn-RhoA-ROCK signaling cascade, through the use of pharmacological or siRNA-mediated inhibition of RhoA-ROCK-II, enhances OPC differentiation in the presence of myelin debris [74]. This is of interest since Fyn directly associates with *α*6*β*1-integrin [12], and signaling from integrins through Fyn modulates oligodendrocyte development via Rho family GTPases [14]. Therefore, myelin debris may inhibit normal functioning of integrin signaling pathways required for remyelination. This finding reveals potential targets for drug development aimed at treating demyelinating diseases.

The above studies implicate the ECM, the integrins, and their downstream pathways in oligodendrocyte-mediated remyelination of white matter tracts. This knowledge will no doubt play an important role in the development of therapeutics for the treatment of demyelinating diseases such as MS.

## 5. Conclusion

Axonal tracts provide an environment rich in molecular cues likely influencing the fate of oligodendrocytes. Specifically, integrin ligation by ECM proteins within axonal tracts can impact oligodendrocyte biology. A specific array of integrin receptors is expressed by oligodendrocytes, and expression may be modulated by extracellular cues. Integrins play a role in the proliferation/survival of oligodendrocytes, as evidenced by reduced survival upon integrin perturbation. Oligodendrocytes experience increased sensitivity to GFs upon ligation of *α*6*β*1-integrin. In some cases, presence of ECM constituents allows the physical interaction of GFRs and *α*6*β*1-integrin, especially in lipid rafts. This interaction between the ECM, integrin receptors, and GFRs mediates oligodendrocyte survival and differentiation with dependence on the PI3K and MAPK signaling pathways. *α*6*β*1-integrin receptors may also interact with non-GFRs to result in parallel cellular responses.

Fyn kinase underlies many of the proliferation/survival effects via integrins in oligodendrocytes, and the same holds true during differentiation, and myelination. *β*1-integrin plays a significant role in the normal development of oligodendrocytes, and depends on specific proteins for signal transduction from the ECM to the cellular interior. In particular, FAK, Akt and Fyn are activated by *β*1-integrins, and may interact with other signaling proteins such as small GTPases.

According to the literature highlighted in this paper, integrins play a major role in the biology of oligodendrocytes. Perhaps in the near future, molecular manipulation of the integrin signaling pathway in oligodendrocytes in a clinical setting will allow cessation of MS disease progression and even promote remyelination of sclerotic plaques.
